# Pachymic Acid Alleviates Non‐Alcoholic Fatty Liver Disease via FGF21‐Mediated Inhibition of the p38 MAPK Pathway

**DOI:** 10.1002/fsn3.71855

**Published:** 2026-05-05

**Authors:** Lijuan Nie, Dehong Ma, Xinke Deng, Aga Erbu, Fanli Wang, Wenshuo Shi, Zixuan Li, Huantian Cui, Weibo Wen

**Affiliations:** ^1^ Medical School Xizang University Lhasa China; ^2^ First School of Clinical Medicine Yunnan University of Chinese Medicine Kunming China

**Keywords:** FGF21, hepatic steatosis, non‐alcoholic fatty liver disease (NAFLD), p38MAPK, pachymic acid (PAC)

## Abstract

Non‐alcoholic fatty liver disease (NAFLD) is characterized by the accumulation of fat in the liver (hepatic steatosis). Pachymic acid (PAC) has demonstrated potential in alleviating hepatic steatosis in NAFLD. However, the molecular mechanisms by which PAC exerts its effects on NAFLD remain unclear. This study aims to elucidate the molecular pathways through which PAC mitigates hepatic steatosis in NAFLD. A free fatty acid (FFA)‐induced hepatic steatosis model was established to assess the regulatory effects of PAC on lipid metabolism. Transcriptome sequencing was performed to explore how PAC influences gene expression in the steatotic HepG2 cells. To confirm the mechanistic role of PAC in hepatic steatosis, we inhibited FGF21 expression in HepG2 cells using nAbFGF21. Finally, a NAFLD mouse model inducted with a high‐fat diet (HFD) was established to further examine PAC's therapeutic effects and underlying pharmacological mechanisms in the context of NAFLD. Research findings showed that PAC treatment significantly reduced lipid accumulation and mitigated cellular damage. Transcriptomic analysis revealed a marked enrichment of the MAPK signaling pathway. Further assays confirmed that PAC increased FGF21 and FGFR1 levels in FFA‐induced HepG2 cells and reduced the phosphorylation of p38MAPK. Importantly, FGF21 emerged as a critical mediator of PAC's pharmacological effects, as neutralizing FGF21 diminished PAC's ability to inhibit p38MAPK activation. In vivo experiments corroborated PAC's therapeutic effects, showing improvements in lipid metabolism and liver injury in NAFLD. Overall, PAC alleviates hepatic steatosis in NAFLD by enhancing FGF21 expression, activating the FGF21/FGFR1 signaling axis, and inhibiting p38MAPK activation.

## Graphical abstract

Pachymic acid alleviates non‐alcoholic fatty liver disease via FGF21‐mediated inhibition of the p38 MAPK pathway. Pachymic acid (PAC) exerts anti‐ non‐alcoholic fatty liver disease (NAFLD) effects by promoting FGF21 expression, activating FGF21/FGFR1 signaling, and thereby suppressing p38 MAPK phosphorylation.

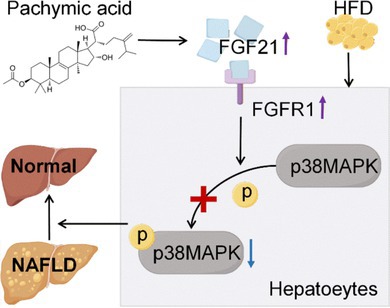

## 1 Introduction

Non‐alcoholic fatty liver disease (NAFLD) is characterized by abnormal fat accumulation in the liver, resulting in hepatic steatosis (Powell et al. 2021). It has become a principal source of chronic liver disease globally (Pouwels et al. 2022). Epidemiological studies estimate the prevalence of NAFLD at approximately 25%–30%, a figure that continues to rise, posing a growing threat to global health (Amini‐Salehi et al. 2024; Younossi et al. 2023). Currently, Resmetirom is the sole drug approved for NAFLD treatment (Feng et al. 2025), however, maintaining a healthy diet and engaging in regular physical activity are the best approaches for managing NAFLD (Zeng et al. 2024). This underscores the pressing need for novel and more effective therapeutic agents to address this widespread condition.

NAFLD is influenced by several factors, including lipid metabolism abnormalities, inflammation, oxidative stress, mitochondrial dysfunction, and hepatocyte damage (Dias et al. 2025; Ma et al. 2016; Pafili et al. 2022). The liver, as the central organ for lipid metabolism, plays a key role in the synthesis, breakdown, and storage of lipids. In the early stages of NAFLD, fat accumulates excessively in the liver, often accompanied by elevated levels of blood lipids (Brunt et al. 2015). Disruptions in hepatic lipid metabolism are fundamental to the development and progression of the disease. Specifically, impaired lipid metabolism hinders fatty acid oxidation, preventing normal lipid breakdown and promoting abnormal lipid buildup in the liver (Grabner et al. 2021). These disturbances not only disrupt normal liver function but also initiate inflammatory responses and contribute to insulin resistance (Rehman and Akash 2016). Together, these interconnected mechanisms drive the progression of liver injury. Therefore, targeting lipid metabolism presents a promising strategy for intervening in NAFLD.

Traditional Chinese Medicine (TCM) emphasizes the regulation of the body's overall balance, a principle that offers distinct advantages in treating complex conditions like NAFLD. Several natural products derived from TCM, such as *Silybum marianum* (Shaker et al. 2025). *S. rebaudiana* root polysaccharide (Bao et al. 2025), Asiaticoside (Wei, Zhang, et al. 2025), and *Cynomorium songaricum* (Liu, Shang, et al. 2025), have shown promising effects in regulating lipid metabolism. Pachymic acid (PAC), a natural compound extracted from the sclerotium of *Wolfiporia cocos*, is one of its key active ingredients. PAC has demonstrated anti‐lipid accumulation and anti‐inflammatory properties (Wei et al. 2022). Recent studies indicate that PAC can reduce hepatic lipid accumulation and liver injury in high‐fat diet (HFD)‐induced NAFLD mice by modulating gut microbiota, lipid metabolism, inflammation, and apoptosis (Ren et al. 2025). However, the precise molecular mechanisms underlying PAC's effects on NAFLD remain unclear and require further investigation.

Here, we first used HepG2 cells and induced hepatic steatosis with free fatty acids (FFA) to assess PAC's regulatory impact on lipid metabolism. We then employed transcriptomic analysis to examine the effect of PAC on gene expression in FFA‐treated HepG2 cells. To further investigate the role of the MAPK signaling pathway in PAC's action on hepatic steatosis, we inhibited FGF21 expression in vitro and established an HFD‐induced NAFLD mouse model in vivo. Our findings offer novel insights into PAC's potential for regulating lipid metabolism in NAFLD and provide a foundation for exploring its pharmacological mechanisms in greater depth.

## 2 Materials and Methods

### 2.1 Experimental Materials

Detailed information on the experimental reagents, pharmaceuticals, assay kits, and other materials used in this study is provided in the Data S1.

### 2.2 In Vitro Experiments

#### 2.2.1 Cell Culture

We obtained human hepatocellular carcinoma HepG2 cells from Shanghai Fuheng Biotechnology Co. Ltd. Cells were maintained at 37°C in a humidified incubator with 5% CO_2_ and Dulbecco's Modified Eagle's Medium (DMEM), which was supplemented with 10% fetal bovine serum (FBS) and 1% penicillin–streptomycin (P/S).

#### 2.2.2 MTT Assay

We seeded HepG2 cells at a density of 1 × 10^4^ cells per well into 96‐well plates. The cells were then treated with 100 μL of DMEM medium serum‐free containing various concentrations of PAC (1.25, 2.5, 5, 10, and 20 μM), while the control group received the same amount of just the medium. After a 24‐h incubation, 10 μL of MTT solution (0.5 mg/mL) was added to each well, and the cells were incubated for an additional 4 h. Following the incubation, the MTT solution was removed, and 100 μL of DMSO was added to dissolve the formazan crystals. A microplate reader was used to measure absorbance at 490 nm, and cell viability was determined accordingly.

#### 2.2.3 Cell Modeling and Experimental Grouping

To establish a cellular model of hepatic steatosis, HepG2 cells were cultured with 1 mM FFA, comprising oleic acid (OA) and palmitic acid (PA) in a 2:1 ratio, for 24 h (Yan et al. 2024). The experimental groups were organized as follows: a normal control group (NC), consisting of cells cultured under standard conditions; a model group (FFA), treated with FFA alone; and a PAC intervention group (FFA+2.5, FFA+5, FFA+10 μM PAC), treated with both FFA and 2.5, 5, 10 μM PAC respectively. Additionally, a drug‐alone intervention group (NC+10 μM PAC) was included for comparison. To assess the impact of PAC on FFA‐induced hepatic steatosis after FGF21 inhibition, we expanded the experimental design to include a positive drug intervention group (FFA+0.1 μM rhFGF21), a neutralizing antibody intervention group (FFA+0.5 μM nAbFGF21), and a combined neutralizing antibody and PAC intervention group (FFA+0.5 μM nAbFGF21 + 10 μM PAC).

#### 2.2.4 Detection of Triglycerides (TG) and FFA

After the treatment period, we collected both the supernatant and cells separately. The FFA concentration in the supernatant was measured using an assay kit. The cells were then washed with phosphate‐buffered saline (PBS) and lysed in RIPA buffer. Following lysis, the samples were centrifuged at 12000 rpm for 10 min, and the supernatant was collected. The TG content in the cell lysates was quantified using a triglyceride assay kit according to the manufacturer's instructions. To normalize the TG levels, the total protein concentration in the cell lysates was measured using the BCA protein assay.

#### 2.2.5 Transcriptome Sequencing

We extracted total RNA from cells in each experimental group using the Trizol reagent, ensuring that the RNA samples met quality criteria for purity, concentration, and integrity. RNA libraries were constructed and sequenced using the Illumina NovaSeq 6000 next‐generation sequencing platform. The sequencing data were then analyzed for differential expressed genes (DEGs) using DESeq2 software.

### 2.3 In Vivo Experiments

#### 2.3.1 Animal

Male C57BL/6 mice aged six‐ to eight‐weeks were obtained from Beijing SiPeiFu Bio‐technology Co. Ltd. (Production License No.: SCXK (Jing) 2024‐0001). We housed mice in groups of five under specific pathogen‐free (SPF) conditions (24°C ± 2°C, 55% ± 5% humidity, and a 12‐h light/dark cycle). They had unrestricted access to food and water throughout the study. This experiment was approved by the Ethical Review Committee of Animal Experiments in Yunnan University of Chinese Medicine and adhered to animal ethics guidelines (Approval No.: YNUCM‐XMSB‐G‐20250274).

#### 2.3.2 Model Establishment, Group Assignment, and Drug Administration

After 1 week of acclimatization, mice were randomly assigned to six groups (*n* = 10 per group): Control, Model, positive drug control (rmFGF21), low‐dose PAC (L‐PAC), medium‐dose PAC (M‐PAC), and high‐dose PAC (H‐PAC). All groups, except the Control, were subjected to NAFLD induction using methods based on previous studies (Pei et al. 2025). The Control group was fed a standard chow diet (10% energy from fat), while the remaining groups received a high‐fat diet (60% fat, 20% protein, 20% carbohydrate) for 12 weeks. During this period, mice in the rmFGF21 group were administered 1.5 mg/kg rmFGF21 via intraperitoneal injection every other day (Wu et al. 2022). Mice in the L‐PAC, M‐PAC, and H‐PAC groups received oral PAC gavage at doses of 10, 25, 50 mg/kg/day (Ma et al. 2015). The Control and Model groups were given equivalent volumes of saline. All mice were fasted for 24 h, anesthetized with 0.3% sodium pentobarbital, and weighed after 12 weeks. Blood samples were collected via the abdominal aorta. Liver tissues were then harvested and weighed in order to calculate the liver index using the following formula:

Liver index

$$\text{Liverindex}\left(\%\right)=\left(\text{Liverweight}/\text{Bodyweight}\right)\times 100$$

#### 2.3.3 Biochemical Parameter Detection

Blood samples were collected and allowed to kepe at room temperature for 30 min, followed by centrifugation at 3000 rpm for 15 min at 4°C. The supernatant was transferred to Eppendorf tubes and centrifuged again at 3000 rpm for 15 min to obtain the final supernatant, which was stored at −80°C. Simultaneously, liver tissues were homogenized, and the homogenates were centrifuged at 3000 rpm for 15 min at 4°C to collect the supernatant. Serum samples were analyzed for TG, total cholesterol (TC), aspartate aminotransferase (AST), and alanine aminotransferase (ALT). The supernatant from liver tissue homogenates was analyzed for TG and TC. All assays were performed according to the manufacturer's instructions provided with the respective kits.

#### 2.3.4 H&E Pathological Staining

Liver tissues were fixed in 4% paraformaldehyde, embedded in paraffin. We sectioned them into 5‐μm slices and dewaxed with xylene. The sections were then rehydrated in a graded ethanol series. Afterward, the tissue sections were stained with hematoxylin and eosin (H&E), cleared with xylene, and mounted with neutral resin. All quantitative analyses were performed using ImageJ.

### 2.4 Oil Red O Staining

Lipid accumulation in HepG2 cells and liver tissues was assessed by Oil Red O staining (Ma et al. 2025). For in vitro experiments, cell slides were rinsed with PBS and fixed in 4% paraformaldehyde at room temperature for 30 min. After two PBS washes, the slides were incubated in 60% isopropanol for 2 min and stained with Oil Red O solution in the dark for 20 min. Excess dye was removed by rinsing 2–5 times with distilled water. Slides were counterstained with Mayer's hematoxylin for 2 min, washed with water, incubated in Oil Red O buffer for 1 min, and mounted. For in vivo experiments, liver tissues were embedded in optimal cutting temperature (OCT) compound, snap‐frozen in liquid nitrogen, and sectioned into 10‐μm slices. Sections were fixed in 4% paraformaldehyde at room temperature for 15 min, and washed with PBS. Sections were stained with Oil Red O solution in the dark for 10 min, followed by sequential washes with 60% isopropanol and PBS (twice each). Nuclei were counterstained with hematoxylin for 5 min, washed three times with PBS, and mounted. Both cell slides and liver sections were examined and photographed under a microscope. The average optical density (AOD) of stained regions was quantified using ImageJ.

### 2.5 ELISA Assay

In vitro, culture supernatants were collected from each group. In vivo, blood samples were collected from all mice, centrifuged at 4000 rpm for 10 min, and the supernatants were used as serum. FGF21 levels in both culture supernatants and serum were determined using ELISA kits per the manufacturer's instructions.

### 2.6 Western Blot Analysis

For each experimental group, protein extracts were prepared from cells or tissues, and protein concentrations were quantified using the BCA assay. We used β‐actin as an internal loading control. Equal amounts of protein were resolved by SDS‐PAGE and transferred onto PVDF membranes using wet transfer. Membranes were blocked with 5% non‐fat milk for 1 h at room temperature and then incubated overnight at 4°C with primary antibodies against FGF21, FGFR1, p‐p38MAPK, and p38MAPK. The next day, membranes were equilibrated to room temperature for 1 h, washed three times with TBST, and incubated for 2 h at room temperature with secondary antibodies. After three additional washes with TBST, protein bands were visualized using ECL reagent. Band intensities were quantified with ImageJ software.

### 2.7 Quantitative Reverse Transcription‐Polymerase Chain Reaction (qRT‐PCR)

Total RNA was isolated from liver tissues of each experimental group using the Trizol reagent method. RNA was then reverse‐transcribed into cDNA with a Takara reverse transcription kit, followed by amplification and quantification by qRT‐PCR. We calculated relative mRNA expression levels using 2^−ΔΔCt^ (Cui et al. 2024). Primer sequences: *Fgf21* (forward primer: 5′‐GCATACCCCATCCCTGACTC‐3′, reverse primer: 5′‐ACCACTGTTCCATCCTCCCT‐3′); *Fgfr1* (forward primer: 5′‐CACCAAACCAAACCCTGTAGC‐3′, reverse primer: 5′‐GGCACTTGAACTTCACCGTC‐3′); *Actb* (forward primer: 5′‐ATATCGCTGCGCTGGTCG‐3′, reverse primer: 5′‐CGATGGAGGGGAATACAGCC‐3′).

### 2.8 Statistical Analysis

Statistical analyses were conducted using SPSS software. Continuous variables that followed a normal distribution were presented as mean ± standard deviation (SD). Differences among multiple groups were assessed by one‐way analysis of variance (ANOVA), followed by post hoc least significant difference (LSD) *t*‐tests for pairwise comparisons. A *p*‐value < 0.05 was considered statistically significant.

## 3 Results

### 3.1 PAC Exhibits Ameliorative Effects on FFA‐Induced Hepatic Steatosis

**FIGURE 1:**
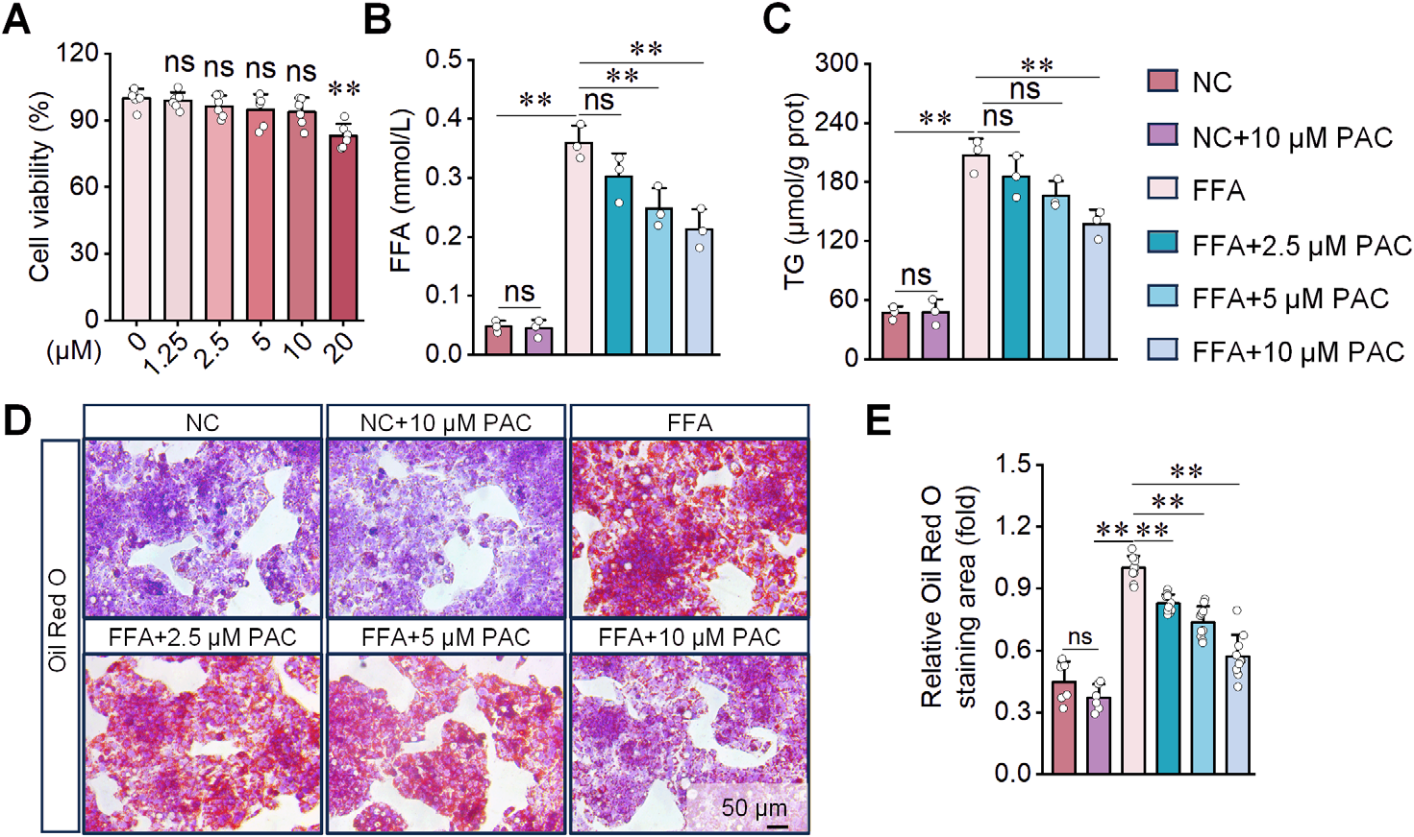
PAC intervention ameliorates FFA‐induced steatosis in HepG2 cells. (A) MTT assay results demonstrating that PAC at concentrations below 10 μM exhibits no significant cytotoxicity in HepG2 cells. (B–E) Analysis of hepatic steatosis‐related parameters in HepG2 cells revealing that PAC intervention dose‐dependently reduces FFA levels in cell supernatants (B) and TG contents within cells (C), while decreases oil red O staining‐positive areas (D, E). *n* = 6 for A; *n* = 3 for B, C. *n* = 10 for (D, E). *p < 0.05, **p < 0.01, ns: not significant.

To assess the effect of PAC on HepG2 cell viability, we treated cells with different PAC concentrations for equal durations. MTT assay results showed that PAC concentrations between 0 and 10 μM did not significantly affect HepG2 cell viability (Figure 1A). Based on these findings, we selected 2.5, 5, and 10 μM PAC for subsequent experiments. To evaluate the effect of PAC on lipid accumulation, we established an FFA‐induced hepatic steatosis model in HepG2 cells and treated cells with PAC. In the FFA group, supernatant FFA levels and intracellular TG content were significantly increased, whereas PAC treatment reduced both parameters to varying degrees (Figure 1B,C). Consistently, cells in the FFA group exhibited extensive lipid droplet accumulation, which was markedly attenuated by PAC (Figure 1D,E). Among the tested concentrations, 10 μM PAC produced the strongest reduction in FFA, TG, and lipid deposition. Therefore, we selected 10 μM PAC for subsequent experiments.

### 3.2 Impact of PAC on Transcriptome of FFA‐Induced HepG2 Cells

**FIGURE 2:**
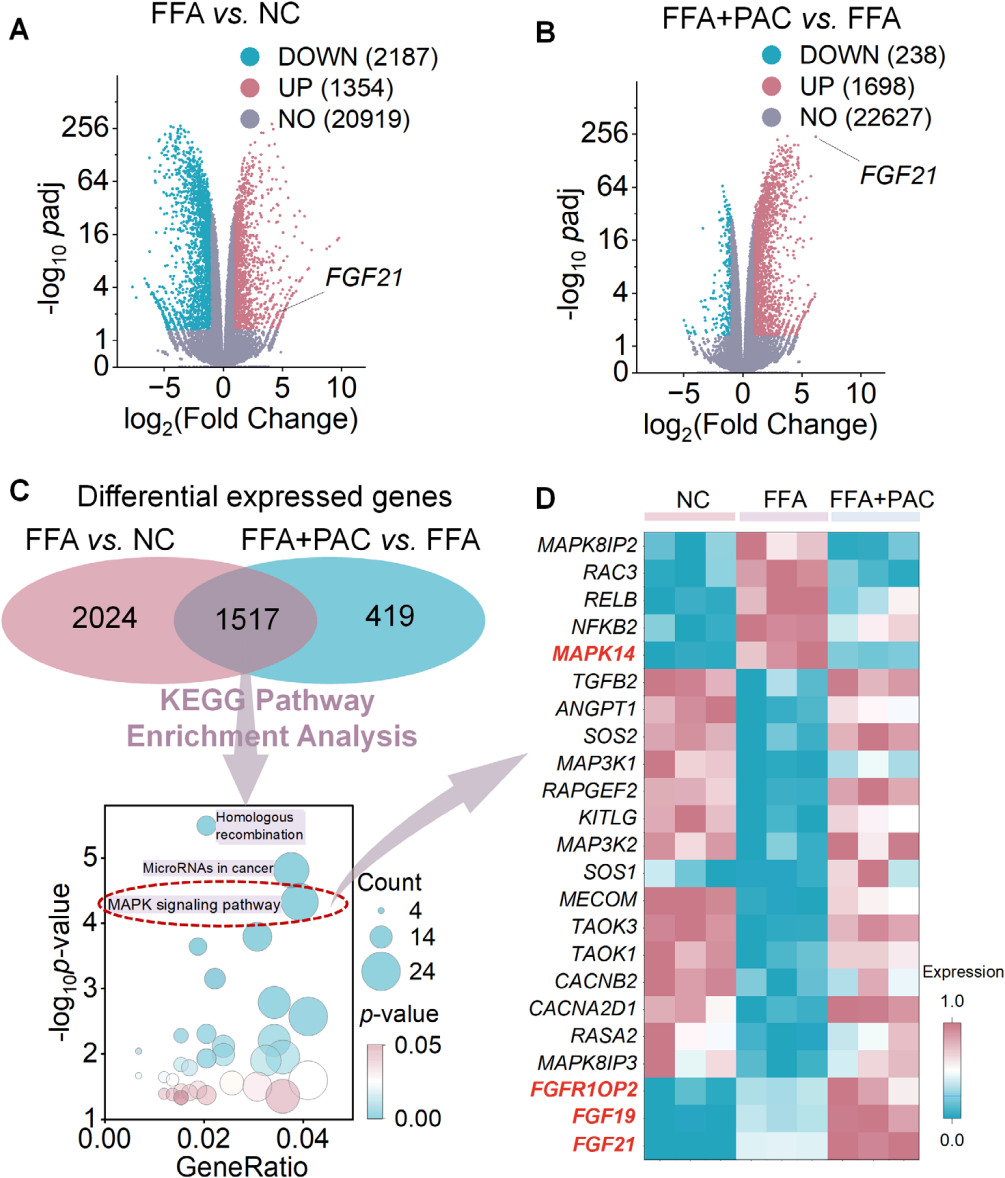
PAC intervention impact on transcriptome of FFA‐induced HepG2 cells. (A, B) Volcano plots visualizing DEGs between FFA versus NC (A) and FFA+10 μM PAC vs. FFA (B). (C) KEGG pathway enrichment analysis conducted on overlapping DEGs identified in both comparisons (FFA vs. NC and FFA+10 μM PAC vs. FFA). Results demonstrated significant enrichment of the MAPK signaling pathway in the KEGG database following PAC treatment. (D) Heatmap displaying expression profiles of genes associated with the MAPK signaling pathway. *n* = 3.

We next conducted transcriptome sequencing of HepG2 cells from the NC, FFA, and FFA+10 μM PAC groups to investigate the effect of PAC on gene expression under FFA‐induced conditions. DEGs were identified using thresholds of *p*_adj_ < 0.05 and |log_2_(fold change)| > 1. Volcano plots illustrate the DEGs profiles (Figure 2A,B). Compared with the NC group, the FFA group exhibited upregulation of 1354 genes and downregulation of 2187 genes (Figure 2A). In contrast, relative to the FFA group, the FFA+10 μM PAC group showed upregulation of 1698 genes and downregulation of 238 genes (Figure 2B). Notably, FGF21 displayed the most pronounced upregulation (highest fold change) after PAC treatment. We next analyzed the overlapping DEGs between the FFA versus NC and FFA+10 μM PAC versus FFA comparisons using Kyoto Encyclopedia of Genes and Genomes (KEGG) pathway enrichment analysis. Applying a significance cutoff of *p‐*value < 0.05, we identified 35 enriched KEGG pathways, with the MAPK signaling pathway emerging as particularly relevant (Figure 2C). The heatmap of genes associated with this pathway (Figure 2D) showed that *FGF19*, *FGF21*, *FGFR1OP2*, and *MAPK14* (*p38MAPK*) were upregulated in the FFA group. After PAC intervention, *FGF19*, *FGF21*, and *FGFR1OP2* remained upregulated, whereas *p38MAPK* expression was markedly suppressed.

### 3.3 PAC Promotes FGF21 Expression While Inhibiting p38MAPK Activation

**FIGURE 3:**
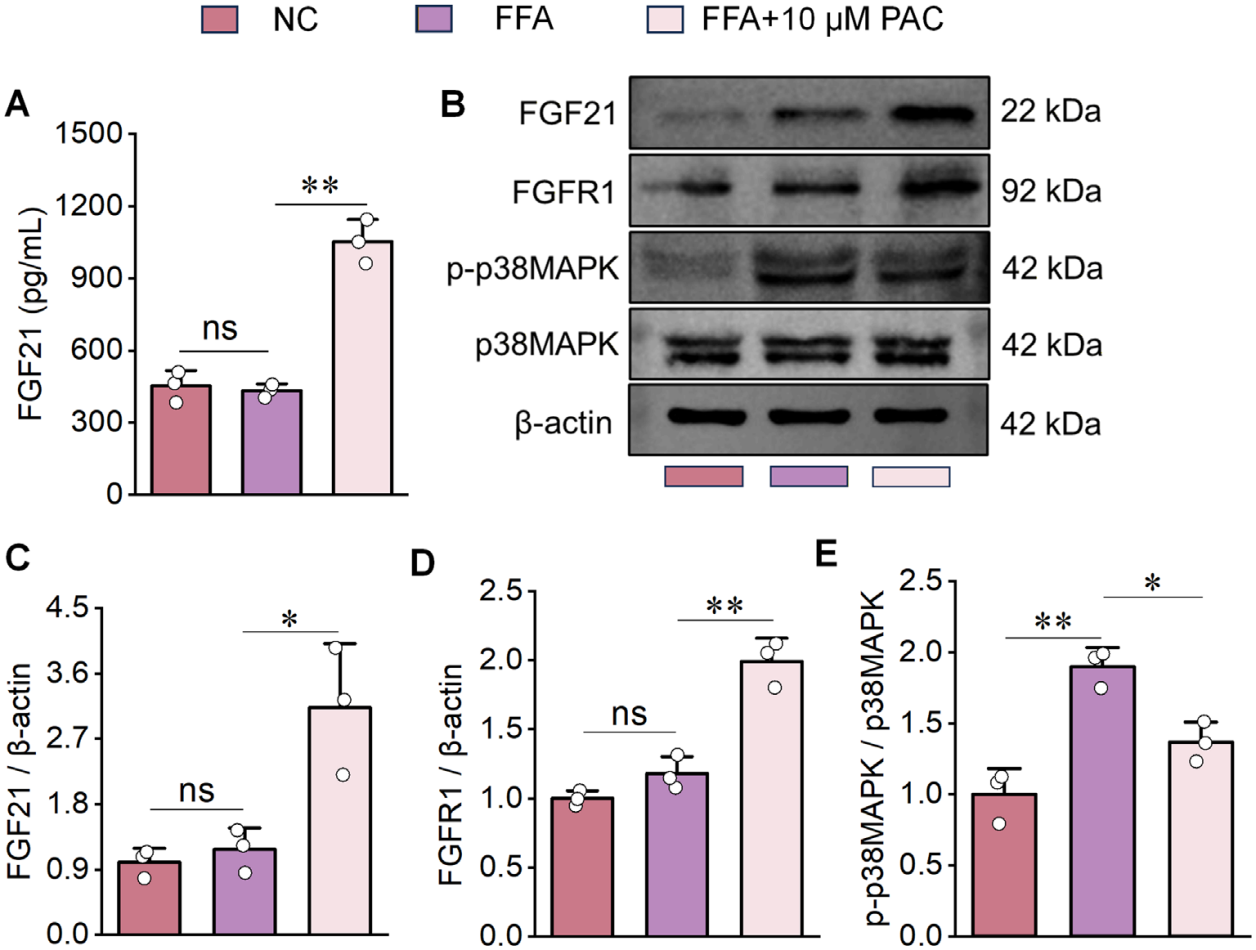
PAC intervention effects on transcriptomic profile of FFA‐induced HepG2 cells. (A) ELISA analysis revealing that PAC intervention increases secretion of FGF21 into the cell supernatant. (B–E) Western blot results demonstrating that PAC significantly upregulates protein expression of FGF21 and FGFR1 but downregulates p‐p38MAPK/p38MAPK in HepG2 cells. *n* = 3.

FGF21 exerts diverse biological functions by binding to FGF receptors (Wang et al. 2025; Xu et al. 2025), with the highest affinity observed for FGFR1 (Qiu et al. 2025). Based on this, we performed additional assays. ELISA results showed that PAC treatment significantly increased FGF21 levels in the cell supernatant of the FFA group (Figure 3A). Western blot analysis further confirmed that PAC upregulated both FGF21 and FGFR1 expression in the FFA group (Figure 3B–D). We also examined p38MAPK phosphorylation. Notably, phosphorylated p38MAPK (p‐p38MAPK) was markedly increased in the FFA group compared with NC controls and significantly reduced after PAC intervention (Figure 3B,E). These results suggest that PAC may alleviate hepatic steatosis by regulating FGF21 expression and p38MAPK activation within the MAPK signaling pathway.

### 3.4 FGF21 Inhibition Attenuates PAC's Ameliorative Effects on Hepatic Steatosis and Abolishes Its Inhibitory Effect on p38MAPK Activation

**FIGURE 4:**
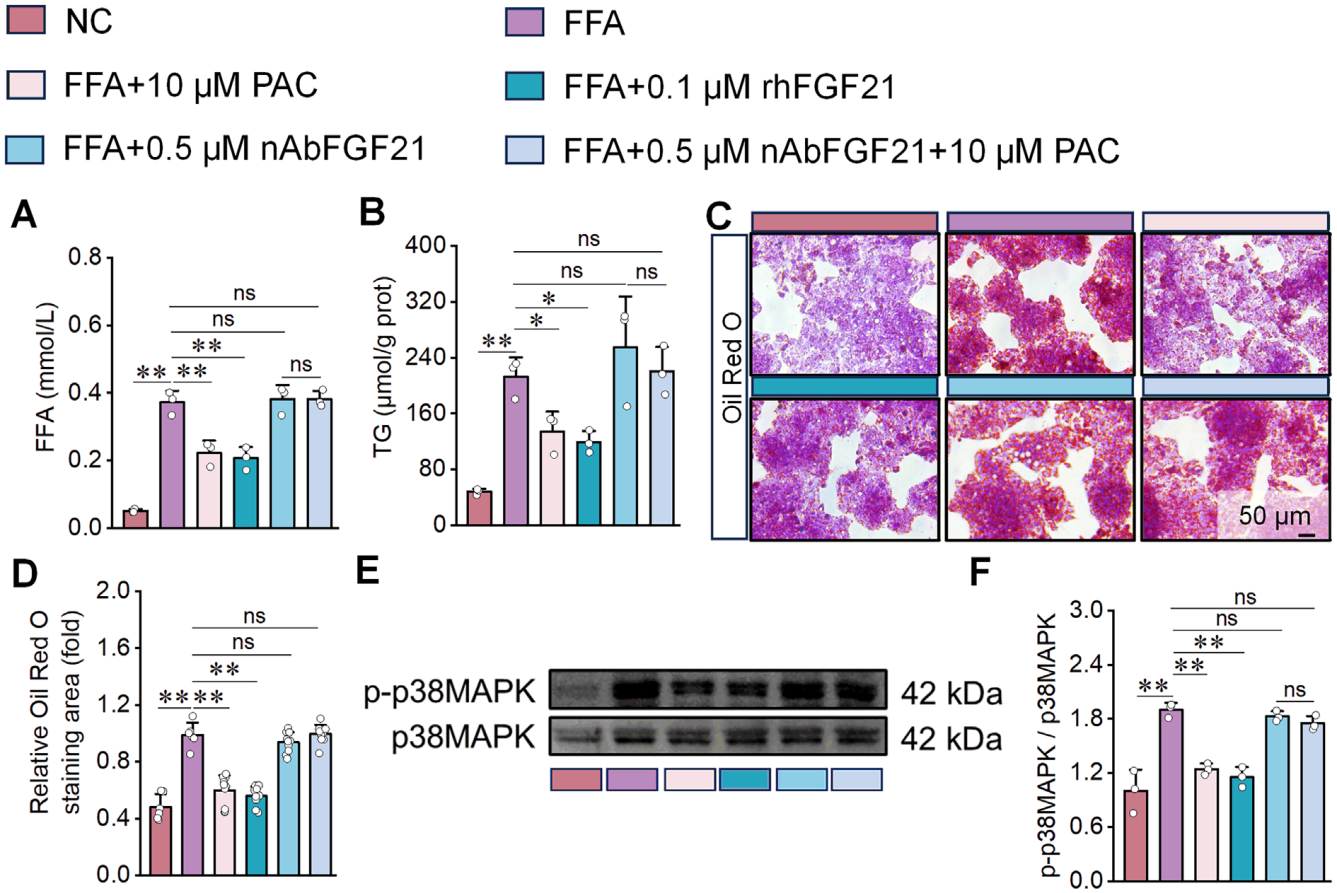
FGF21 neutralization attenuates protective effects of PAC against HepG2 cell steatosis. (A–D) PAC exhibits comparable intervention effects to rhFGF21 in mitigating FFA‐induced steatosis in HepG2 cells as evidenced by reductions in supernatant FFA content (A), intracellular TG levels (B), and oil red O‐positive staining area (C, D). However, these protective effects of PAC are significantly attenuated following FGF21 neutralization. (E, F) Western blot analysis revealing no significant differences in the p38MAPK phosphorylation ratio between FFA+0.5 μM nAbFGF21 + 10 μM PAC and FFA groups. *n* = 3 for (A, B, E, F); *n* = 10 for C, D.

To investigate the mechanism underlying PAC's protective effects on FFA‐induced hepatic steatosis, we conducted synchronized interventions in FFA‐treated HepG2 cells using either an FGF21‐neutralizing antibody (nAbFGF21) or recombinant human FGF21 (rhFGF21). Both the FFA+10 μM PAC and FFA+0.1 μM rhFGF21 groups exhibited significantly reduced FFA levels and TG content in the supernatant compared with the FFA‐only group (Figure 4A,B). Consistently, intracellular lipid accumulation was markedly decreased in these groups (Figure 4C,D), further supporting PAC's robust protective role against FFA‐induced hepatocellular steatosis. In contrast, neutralization of FGF21 substantially attenuated or completely abolished PAC's ameliorative effect. In the FFA+0.5 μM nAbFGF21 + 10 μM PAC group, FFA levels, TG content, and lipid accumulation showed no significant differences compared with the FFA‐only group (Figure 4A–D). Western blot analysis further confirmed that FGF21 neutralization eliminated PAC's inhibitory effect on p38MAPK phosphorylation in FFA‐exposed HepG2 cells, with levels comparable to those of the FFA group (Figure 4E,F).

### 3.5 PAC Exhibits Therapeutic Effects in NAFLD Mice

**FIGURE 5:**
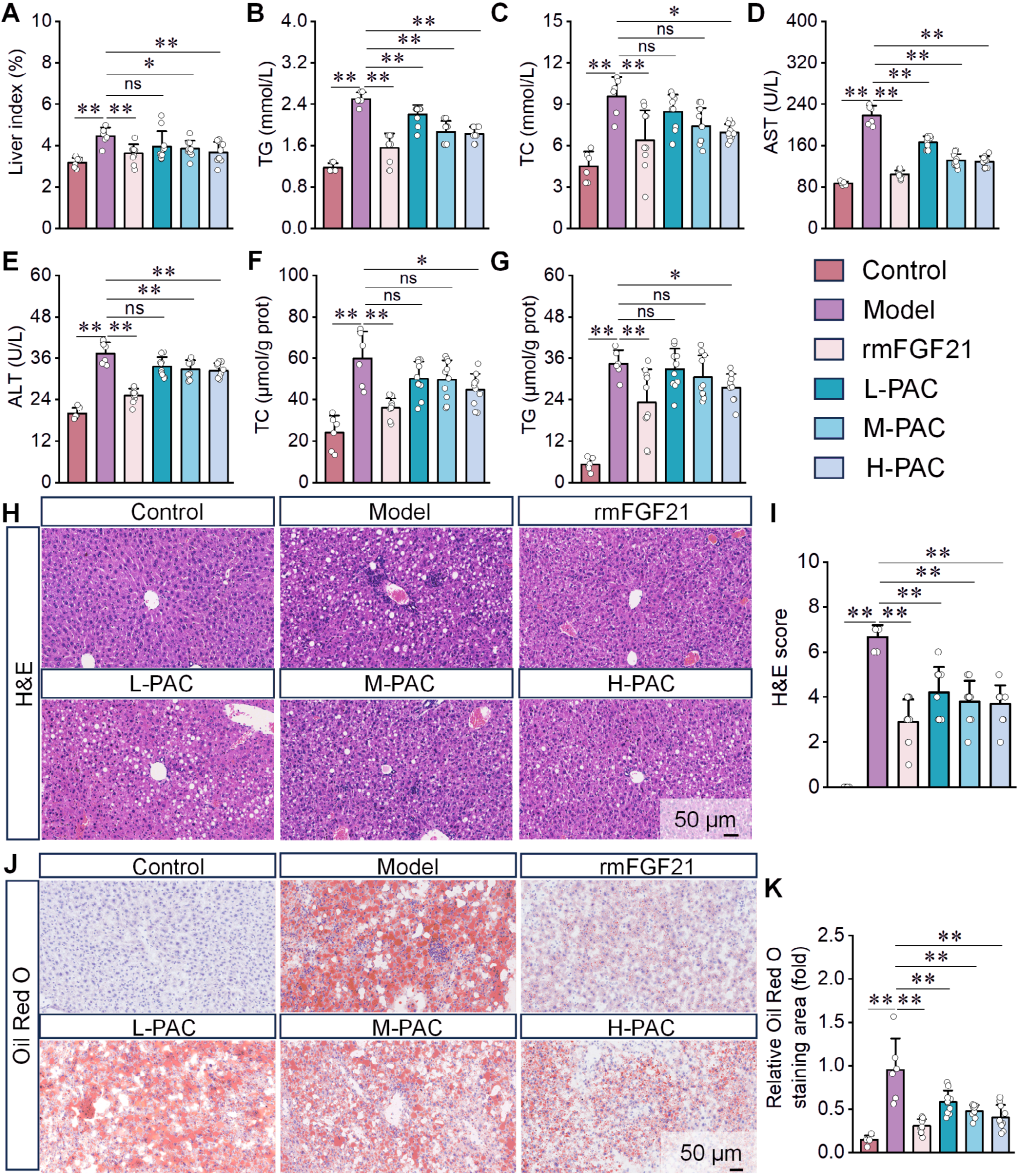
PAC alleviates hepatic injury in NAFLD mice. (A–G) PAC significantly reduces the liver index (A), serum TG (B), TC (C), AST (D), and ALT (E) levels, as well as hepatic tissue TC (F) and TG (G) contents in NAFLD mice. (H, I) HE staining of liver sections demonstrating that PAC intervention attenuates hepatocyte injury in NAFLD mice. (J, K) Oil red O staining revealing that PAC treatment decreases lipid deposition in hepatic tissues of NAFLD mice. *n* = 10 for A–K.

We next evaluated PAC's therapeutic potential in vivo using an HFD‐induced NAFLD mouse model. PAC treatment significantly reduced hepatic index, serum TG, TC, AST, and ALT, as well as hepatic TG and TC levels in NAFLD mice (Figure 5A–G). Histological analysis by H&E staining revealed characteristic pathological changes in NAFLD livers, including hepatocyte enlargement, cytoplasmic vacuolization, nuclear margination, and inflammatory cell infiltration (Figure 5H,I). Oil Red O staining further confirmed extensive red lipid droplet deposition with markedly increased numbers in NAFLD livers (Figure 5J,K). PAC administration alleviated these pathological features to varying degrees, confirming its hepatoprotective effects. Remarkably, high‐dose PAC produced the strongest improvement, with efficacy comparable to recombinant murine FGF21 (rmFGF21).

**FIGURE 6:**
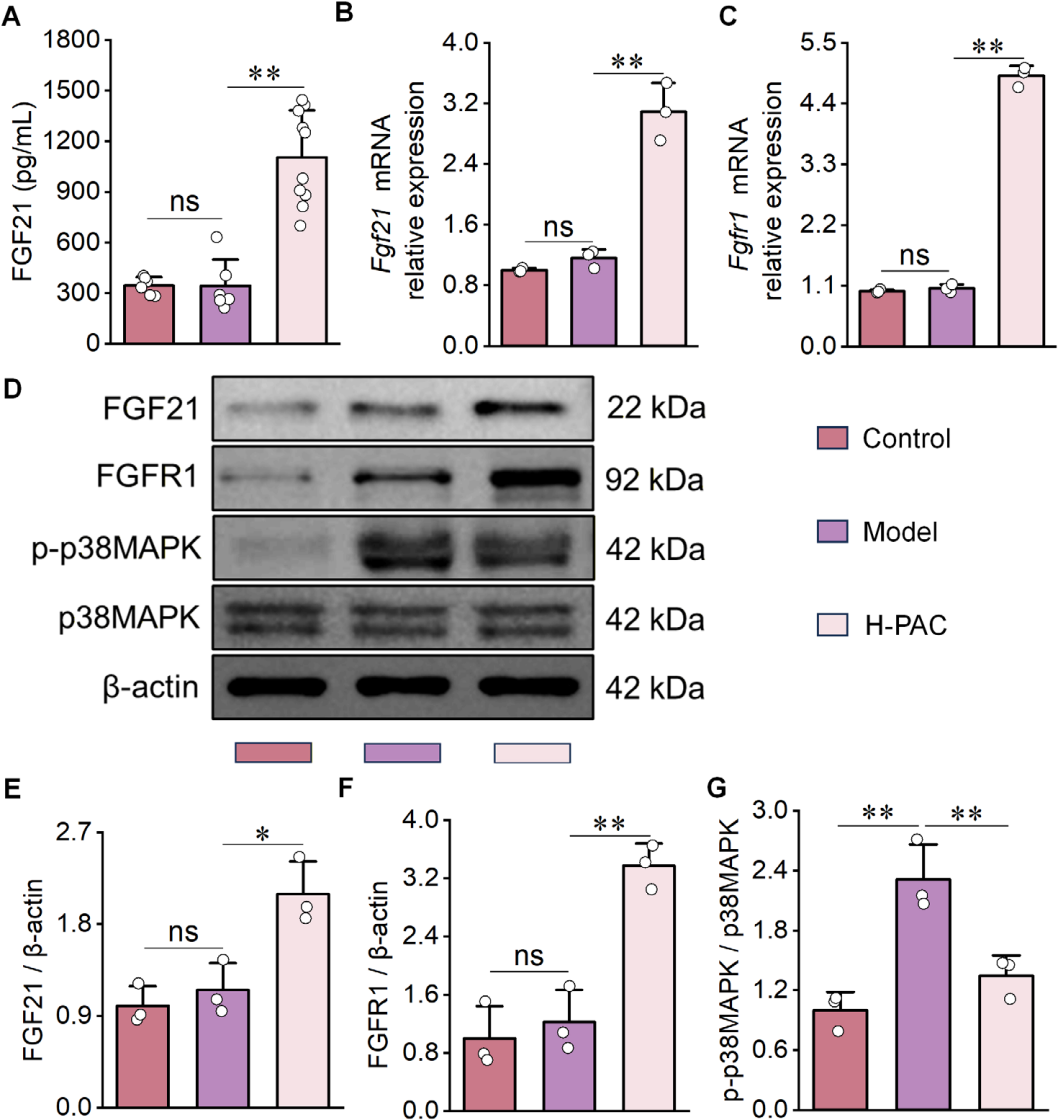
Validation of regulatory effects of PAC on MAPK signaling pathway‐related factors in NAFLD mice. (A) ELISA analysis revealing that PAC intervention significantly elevates serum fibroblast growth factor FGF21 levels in NAFLD mice. (B, C) qRT‐PCR demonstrating that PAC treatment upregulates hepatic *Fgf21* and *Fgfr1* mRNA expression in NAFLD mice. (D–G) Western blot analysis of liver tissues showing that PAC intervention increases protein expression of FGF21 (D, E) and FGFR1 (D, F) and decreases the phosphorylation ratio of p38 MAPK (D, G) in NAFLD mice. *n* = 10 for A; *n* = 3 for B–G.

We further investigated the role of the MAPK signaling pathway in this process. Serum ELISA analysis revealed elevated FGF21 levels in NAFLD mice, which remained high following PAC treatment (Figure 6A). qRT‐PCR analysis showed that PAC increased hepatic expression of *Fgf21* and *Fgfr1* (Figure 6B,C). Consistently, Western blot results demonstrated significantly elevated protein levels of FGF21 and FGFR1 (Figure 6D–F), along with a reduction in the p38MAPK phosphorylation ratio in PAC‐treated NAFLD livers (Figure 6D,G). These findings mirror the in vitro results and suggest that PAC ameliorates NAFLD by enhancing FGF21/FGFR1 signaling to suppress p38MAPK activation.

## 4 Discussion

NAFLD is strongly associated with dietary changes, particularly excessive intake of saturated fats and fructose combined with reduced fiber consumption. These lifestyle factors have contributed to a steady rise in NAFLD incidence worldwide, with cases increasingly reported in younger populations (Bao et al. 2025). NAFLD is now recognized as the most prevalent chronic liver disease globally (Zhang et al. 2016). In the absence of pharmacological intervention, NAFLD can progress to nonalcoholic steatohepatitis (NASH) and eventually hepatocellular carcinoma (HCC) (Bao et al. 2025). Despite its growing burden, effective therapeutic options for NAFLD remain critically limited (EASL et al. 2024), resulting in poor clinical outcomes and reduced quality of life for affected patients. TCM and its bioactive compounds have long been investigated as potential treatments for NAFLD (Alshehade et al. 2022; Hsu et al. 2016; Shaker et al. 2025), offering valuable insights for the development of novel therapeutic strategies. *Wolfiporia cocos*, the dried sclerotium of the polypore fungus Poria cocos, holds an important place in TCM due to its broad pharmacological activities (Deng et al. 2025; Lei et al. 2025; Liu, Yu, et al. 2025). Among its unique bioactive constituents, PAC has shown promising efficacy in alleviating hepatic steatosis in NAFLD mouse models (Ren et al. 2025).

In this study, we first assessed PAC's effects in an in vitro model of FFA‐induced cellular steatosis, which is widely used to evaluate drug activity against hepatic lipid accumulation—a hallmark of NAFLD pathology (Fan et al. 2025; Ma et al. 2025). Our results demonstrated that PAC markedly reduced intracellular FFA and TG levels while suppressing lipid droplet deposition in FFA‐treated HepG2 cells, supporting its therapeutic potential in mitigating steatosis.

We next performed transcriptomic sequencing of HepG2 cells from different experimental groups to clarify the molecular mechanisms through which PAC alleviates hepatic steatosis. DEGs analysis revealed robust upregulation of fibroblast growth factor 21 (FGF21) following PAC treatment, prompting a more focused investigation of this pathway. FGF21, first identified in 2005 as a member of the fibroblast growth factor (FGF) family, has emerged as a pivotal metabolic regulator (Kharitonenkov et al. 2005). It is primarily secreted by the liver into circulation (Wang et al. 2025), where it binds fibroblast growth factor receptors (FGFRs), with the highest affinity for FGFR1, to promote lipid and glucose metabolism. Through these actions, FGF21 lowers serum TG and cholesterol levels (Chen et al. 2023; Negroiu et al. 2024; Qiu et al. 2025). Elevated hepatic expression of FGF21 has been consistently reported in metabolic disorders such as NAFLD, obesity, and type 2 diabetes mellitus (T2DM) (Gliniak et al. 2025; Wei, Zhang, et al. 2025; Zhang et al. 2025). Moreover, high‐carbohydrate diets further stimulate hepatic FGF21 production (Negroiu et al. 2024), whereas exercise and dietary interventions enhance FGF21 expression, thereby improving lipid metabolic dysfunction (Gao et al. 2020). Together, these findings support a central role for FGF21 as a regulator in the development and progression of metabolic diseases. Clinical studies have further highlighted the therapeutic promise of FGF21. Several trials using FGF21 analogs, including PF‐05231023 (Talukdar et al. 2016) and pegbelfermin (Loomba et al. 2024), have demonstrated improvements in hepatic lipid metabolism, glycemic control, and insulin resistance in patients. In parallel, numerous animal studies have shown that natural compounds such as *theabrownin* (Zhen et al. 2023), *wogonin* (Yamada et al. 2022), and *hesperidin* (Aja et al. 2022) increase FGF21 expression, alleviating hepatic metabolic dysfunction. Conversely, liver‐specific knockout of FGFR1 in mice significantly decreased serum TG, FFA, and hepatic FFA levels (Adams et al. 2012), underscoring the essential role of FGF21/FGFR1 signaling in lipid homeostasis.

KEGG pathway enrichment analysis identified MAPK signaling pathway as significantly enriched. Within this pathway, several key DEGs—including fibroblast growth factor 19 (FGF19), FGF21, and FGFR1 oncogene partner 2 (FGFR1OP2)—were strongly upregulated following PAC treatment, whereas p38 mitogen‐activated protein kinase (p38MAPK) expression was markedly reduced. FGF19, an intestinal hormone structurally related to FGF21, is a critical regulator of hepatic lipid homeostasis (Lopez‐Pascual et al. 2024). Both FGF19 overexpression and pharmacological administration have been shown to reverse pathological features of fatty liver disease, obesity, and hyperlipidemia (Li et al. 2024). In contrast, NAFLD activates p38MAPK, leading to increased phosphorylation (p‐p38MAPK). Elevated p‐p38MAPK suppresses fatty acid oxidation, thereby aggravating intracellular lipid accumulation and driving NAFLD progression (Su et al. 2025; Wu et al. 2022; Zhang et al. 2016). Numerous in vitro and in vivo studies confirm that inhibition of p38MAPK phosphorylation alleviates lipid metabolic dysfunction (Jiang et al. 2019; Liu et al. 2018). FGFR1OP2, a partner protein of FGFR1, contributes to cellular proliferation and repair (Wang et al. 2024). Its upregulation following PAC treatment suggests possible hepatoprotective and regenerative roles, although its direct involvement in lipid metabolism remains unclear and requires further investigation. Given the central importance of hepatic lipid metabolism in this study, we prioritized validation of FGF21, FGFR1, and p38MAPK. Our results demonstrated that PAC treatment significantly increased protein expression of FGF21 and FGFR1 while suppressing p38MAPK phosphorylation. These findings provide strong preliminary evidence that PAC regulates FFA‐induced hepatic steatosis. Specially, we observed FFA‐induced upregulation of FGF21 mRNA, but no corresponding increase in the supernatant. The discrepancy between FGF21 expression at the transcriptional and secretory levels may be related to post‐translational or secretory pathway regulation. Previous study has demonstrated that although both PA and OA could upregulate FGF21 mRNA expression, only OA effectively promoted FGF21 protein secretion, whereas PA failed to induce secretion (Mai et al. 2009). In our study, hepatic steatosis was induced using FFA mixture (OA:PA = 2:1). Following 24 h of treatment, no significant increase in FGF21 protein was detected in the supernatant, suggesting that PA may have overridden the secretion‐promoting effect of OA in this mixed system. Elucidating the differential regulatory mechanisms of saturated versus unsaturated fatty acids on FGF21 secretion will provide deeper insights into NAFLD pathophysiology.

Our findings indicate that PAC's protective effects against FFA‐induced hepatic steatosis are closely linked to modulation of FGF21 and p38MAPK within the MAPK signaling pathway. Previous studies have shown that blocking FGF21/FGFR1 signaling reduces p38MAPK phosphorylation, particularly when FGF21 expression is elevated by pharmacological intervention (Wu et al. 2022). We therefore propose that PAC enhances FGF21 expression, activates FGF21/FGFR1 signaling, and then suppresses p38MAPK phosphorylation to alleviate FFA‐induced steatosis. To test this hypothesis, we treated FFA‐stimulated HepG2 cells with nAbFGF21 (Hu et al. 2024), using rhFGF21 (Li et al. 2023; Ye et al. 2019) as a positive control. Our results show that FGF21 neutralization abolished PAC's regulatory effects on the pathway, supporting our proposed mechanism.

We next assessed PAC's therapeutic potential in vivo using an HFD‐induced NAFLD mouse model (Jiang et al. 2025). PAC treatment significantly improved NAFLD pathology by reducing hepatic inflammatory infiltration, lipid accumulation, and liver injury, while restoring lipid metabolic balance. These results were consistent with our in vitro findings, confirming PAC's anti‐NAFLD activity. Furthermore, PAC's effects on MAPK pathway components—FGF21, FGFR1, and p‐p38MAPK—in vivo paralleled those observed in vitro. Of note, PAC exhibited a therapeutic profile similar to rmFGF21, a validated NAFLD treatment that markedly reduces hepatic injury (Kim and Yoo 2022), thereby reinforcing PAC's modulation of the MAPK signaling pathway in a physiological context.

Our study has investigated the mechanism of action of PAC and its potential as a therapeutic candidate for NAFLD. However, the present study has several limitations. First, only three samples were used for some experiments, including transcriptome sequencing and western blotting. The small sample size may compromise statistical reliability and the power to detect differential expression. A large sample size of sequencing analysis, functional verification, and clinical trials will be conducted in future studies, further promoting PAC's clinical translation. Second, although an association has been established between PAC‐mediated inhibition of p38MAPK activation and enhanced FGF21/FGFR1 signaling, the direct target by which PAC alleviates hepatic steatosis in NAFLD remains to be identified. Integration of molecular docking, drug affinity responsive target stability (DARTS), surface plasmon resonance (SPR), and bio‐layer interferometry (BLI) assays will enable validation of PAC's direct target.

## 5 Conclusions

In conclusion, our study shows that PAC has beneficial effects in alleviating hepatic steatosis in NAFLD. Concretely, PAC promotes FGF21 expression within the MAPK signaling pathway, activates FGF21/FGFR1 signaling, and subsequently inhibits p38MAPK activation. These results suggest that PAC may serve as a potential therapeutic agent for the prevention and treatment of NAFLD.

## Author Contributions

**Wenshuo Shi:** investigation. **Fanli Wang:** software, investigation. **Lijuan Nie:** writing – original draft, conceptualization, funding acquisition. **Dehong Ma:** writing – original draft, data curation, formal analysis. **Weibo Wen:** project administration, writing – review and editing, funding acquisition. **Huantian Cui:** conceptualization, writing – review and editing. **Zixuan Li:** project administration, writing – review and editing, funding acquisition. **Xinke Deng:** formal analysis, data curation, investigation. **Aga Erbu:** formal analysis, data curation.

## Funding

This work was supported by the National Natural Science Foundation of China (No. 82560899, China), Lhasa Science and Technology Planning Projects (No. LSKJ202444, China) and Science and Technology Projects of Xizang Autonomous Region (No. XZ202402ZY0002, China).

## Conflicts of Interest

The authors declare no conflicts of interest.

## Supporting information


**Data S1:** Supporting Information.

## Data Availability

The data that support the findings of this study are available from the corresponding author upon reasonable request.
