# Supplementary material for: Pachymic Acid Alleviates Non‐Alcoholic Fatty Liver Disease via FGF21‐Mediated Inhibition of the p38 MAPK Pathway
Source: Food Sci Nutr. 2026 May 5;14(5):e71855. doi: 10.1002/fsn3.71855 (PMC13144552; doi:10.1002/fsn3.71855)
Supplement: Supplementary file 1 — Data S1: Supporting Information. [file FSN3-14-e71855-s001.docx]

**Supplementary materials**

**1 Reagents**

High-fat diet chow was purchased from SPF (Beijing) Biotechnology Co., Ltd. (Beijing, China). Pachymic acid (C_33_H_52_O_5_, CAS: 29070-92-6), FGF-21 Protein (HY-P70473) were purchased from MedChemExpress (Shanghai, China). Non-esterified fatty acids (NEFA, A042-2-1) and Triglyceride (TG, A110-1-1), Alanine aminotransferase (ALT, C009-2-1) and aspartate aminotransferase (AST, C010-2-1) were purchased from Nanjing Jiancheng Biological Engineering Institute (Nanjing, China). H&E staining kit (G1120) and Oil Red O Staining Kit (G1262) were purchased from Solarbio Science & Technology Co., Ltd. (Beijing, China). Human FGF21 ELISA Kit (ab222506), Anti-FGF21 (ab171941) and Anti-FGFR1 (ab59194) were purchased from Abcam (Shanghai, China). Mouse FGF21 ELISA (RD291108200R) was purchased from Biovendor Research and Diagnostic Products (Wuhan, China). p38 MAPK Antibody (9212S) and Phospho-p38 MAPK (4511S) were purchased from Cell Signaling Technology, Inc. (Shanghai, China). Recombinant Mouse FGF21 Protein (8409-FG), FGF21 neutralizing antibody (AF3057) were purchased from R&D Systems, Inc. (Shanghai, China).
